# Cardiac sarcoidosis: diagnosis and management

**DOI:** 10.3389/fcvm.2024.1394075

**Published:** 2024-10-08

**Authors:** Abdullah Aftab, Stanley Szeto, Zoha Aftab, Sabahat Bokhari

**Affiliations:** ^1^ Department of Internal Medicine, Montefiore Medical Center, New York, NY, United States; ^2^ Department of Cardiology, Robert Wood Johnson University Hospital, New Brunswick, NJ, United States; ^3^ Norwich Medical School, University of East Anglia, Norwich, United Kingdom

**Keywords:** cardiac sarcoidosis, Cardiac Magnetic Resonance Imaging, late gadolinium enhancement, positron emission tomography, inflammatory heart disease

## Abstract

Non-caseating granulomatous infiltration of the myocardium is the hallmark of cardiac sarcoidosis (CS). CS manifests clinically as conduction disturbance, ventricular arrhythmia, sudden cardiac death and/or heart failure with reduced ejection fraction. Other than confirmation through endomyocardial biopsy, a diagnosis of probable CS can be established by histological evidence of systemic sarcoidosis in addition to characteristic clinical or advanced imaging findings. Cardiac Magnetic Resonance imaging (CMR) and ^18^F-flurodeoxyglycose positron emission tomography (FDG-PET) are imaging modalities indispensable in the diagnosis and monitoring of CS. FDG-PET is the method of choice for identifying the active inflammatory phase of CS and in the monitoring and modifying of immunosuppressive treatment. CMR is better suited for assessing cardiac morphology and function. Both modalities are more effective in detecting CS when used in combination than either is alone. Management of CS is primarily based upon observational data of low quality due to a paucity of randomized controlled trials. Corticosteroid therapy and/or tiered-immunosuppression are the mainstays of treatment in reducing myocardial inflammation. Steroid-sparing agents aim to limit the unfavorable side-effects of a significant steroid burden. Antiarrhythmics and guideline-directed medical therapies are utilized for control of ventricular arrhythmia and left ventricular dysfunction respectively. CS necessitates multidisciplinary care in specialized centers to most effectively diagnose and manage the disease. Additional randomized trials are warranted to further our understanding of medical optimization in CS.

## 1 Introduction

Sarcoidosis is a multisystem granulomatous inflammatory disorder of unknown etiology. It is characterized typically by the presence of non-caseating granulomas that may develop in any part of the body, resulting in scarring and fibrosis or spontaneous resolution (1). Cardiac sarcoidosis (CS) is an infiltrative cardiomyopathy that presents concurrently in 20%–27% of patients with extracardiac disease or rarely as an isolated occurrence (2, 3). Isolated cardiac disease portends a poorer prognosis than CS with extracardiac involvement (4); patients with isolated CS suffer from worse LV systolic function at presentation and a greater burden of ventricular tachycardia (3). CS can manifest itself as aberrant atrioventricular conduction disturbances, ventricular arrhythmia or even sudden cardiac death (2). There are various criteria employed to confirm a clinical diagnosis of CS, however, none are validated or have garnered universal adoption (5–8) (Tables 1, 2). There currently exists a degree of uncertainty surrounding CS, influenced by factors such as the undetermined etiology of the disease, difficulties in establishing the presence of myocardial granuloma, and a lack of randomized controlled trials and personalized therapeutics. This article will review and elucidate the clinical diagnosis and management of CS.

**Table 1:** Guidelines for diagnosis of cardiac sarcoidosis based on the 2006 revised guidelines of the Japanese society of sarcoidosis and other granulomatous disorders.

Japanese society of sarcoidosis and other granulomatous disorders (2006)	
Histological diagnosis group	Clinical diagnosis group
Endomyocardial biopsy specimens demonstrate noncaseating epithelioid cell granulomas with histological or clinical diagnosis of extracardiac sarcoidosis	Extracardiac sarcoidosis is diagnosed histologically or clinically and satisfies either of the following conditions: - ≥2 of the MAJOR criteria - 1 in 4 of the MAJOR criteria and ≥5 minor criteria
	Major criteria: - Advanced AV Block - Basal thinning of the interventricular septum - Positive ^67^Gallium uptake in the heart - Depressed ejection fraction of the ventricle (<50%)
	Minor criteria: - Abnormal ECG: ventricular arrhythmias, complete RBBB, abnormal axis, abnormal Q wave - Abnormal echocardiogram: regional wall motion or morphological abnormality - Nuclear medicine: perfusion defect by ^201^TI or ^99m^Tc myocardial scintigraphy - Gadolinium-enhanced CMR: delayed enhancement of myocardium - Endomyocardial biopsy: interstitial fibrosis or monocyte infiltration over moderate grade

**Table 2:** HRS expert consensus statement on the diagnosis and management of arrhythmias associated with cardiac sarcoidosis.

Heart rhythm society expert consensus statement (2014)
It is probable that CS is present if all 3 of the following conditions are met:
(a) There is a histological diagnosis of extracardiac sarcoidosis (b) One or more of the following is present: • Corticosteroid or immunosuppressant- responsive cardiomyopathy or heart block • Unexplained reduced LV ejection fraction (40%) • Unexplained sustained (spontaneous or induced) ventricular tachycardia, Mobitz type II second-degree heart block or third-degree heart block • Patchy uptake (of FDG) on dedicated cardiac PET in a pattern consistent with CS • Late gadolinium enhancement on CMR in a pattern consistent with CS • Positive gallium uptake in a pattern consistent with CS (c) Other causes for the cardiac manifestation(s) have been reasonably excluded

## 2 Diagnosis

The diagnosis of CS necessitates a multi-pronged approach involving histological evidence, exclusion of other diagnoses, and the presence of particular clinical features. The histological diagnosis of definite CS can be made from endomyocardial biopsy demonstrating non-caseating granuloma without an alternative cause, as per the HRS consensus statement. The sensitivity of endomyocardial biopsy is poor however due to patchy myocardial involvement (9, 10) ranging from 25%–36%; this can be improved to 50% if intracardiac voltage mapping, ^18^F-flurodeoxyglycose positron emission tomography (FDG-PET), or Cardiac Magnetic Resonance imaging (CMR) guided biopsy is performed (9, 11). Given these limitations, diagnostic criteria for probable CS were formulated which include extracardiac histological identification of sarcoid and the presence of one of the following clinical characteristics not explained by other etiologies (Table 1). These clinical characteristics include ejection fraction less than 40%, sustained ventricular tachycardia, Mobitz type II or complete heart block, FDG-PET demonstrating patchy uptake, CMR showing late gadolinium enhancement or gallium scintigraphy showing positive gallium uptake (6).

Typical CS symptomatology that should necessitate further workup includes chest pain, palpitations, and (pre)syncopal episodes. While diagnostic and prognostic biomarkers have not yet been established for CS, serum B-type natriuretic peptide levels have proven to be a useful diagnostic marker for cardiac involvement in systemic sarcoidosis; cardiac troponin I was also shown to be a predictor of fatal arrhythmia in CS patients in a single study (12, 13). Electrocardiography and cardiac event monitoring can aid in the detection of atrioventricular conduction disturbances and ventricular tachycardia in patients who present with palpitations. Traditional echocardiographic parameters do not show findings sensitive or specific for CS in early disease as focal myocardial involvement is usually too small to detect (9, 14). Two-dimensional–speckle tracking echocardiography is a more sensitive technique and can predict subclinical myocardial involvement in CS patients by way of left and right ventricular global longitudinal strain measurement (15). Typical echocardiographic findings of more advanced disease include LV dilation with systolic dysfunction, regional wall motion abnormalities in a noncoronary distribution, septal wall thinning and ventricular aneurysm formation (16).

**Figure 1:**
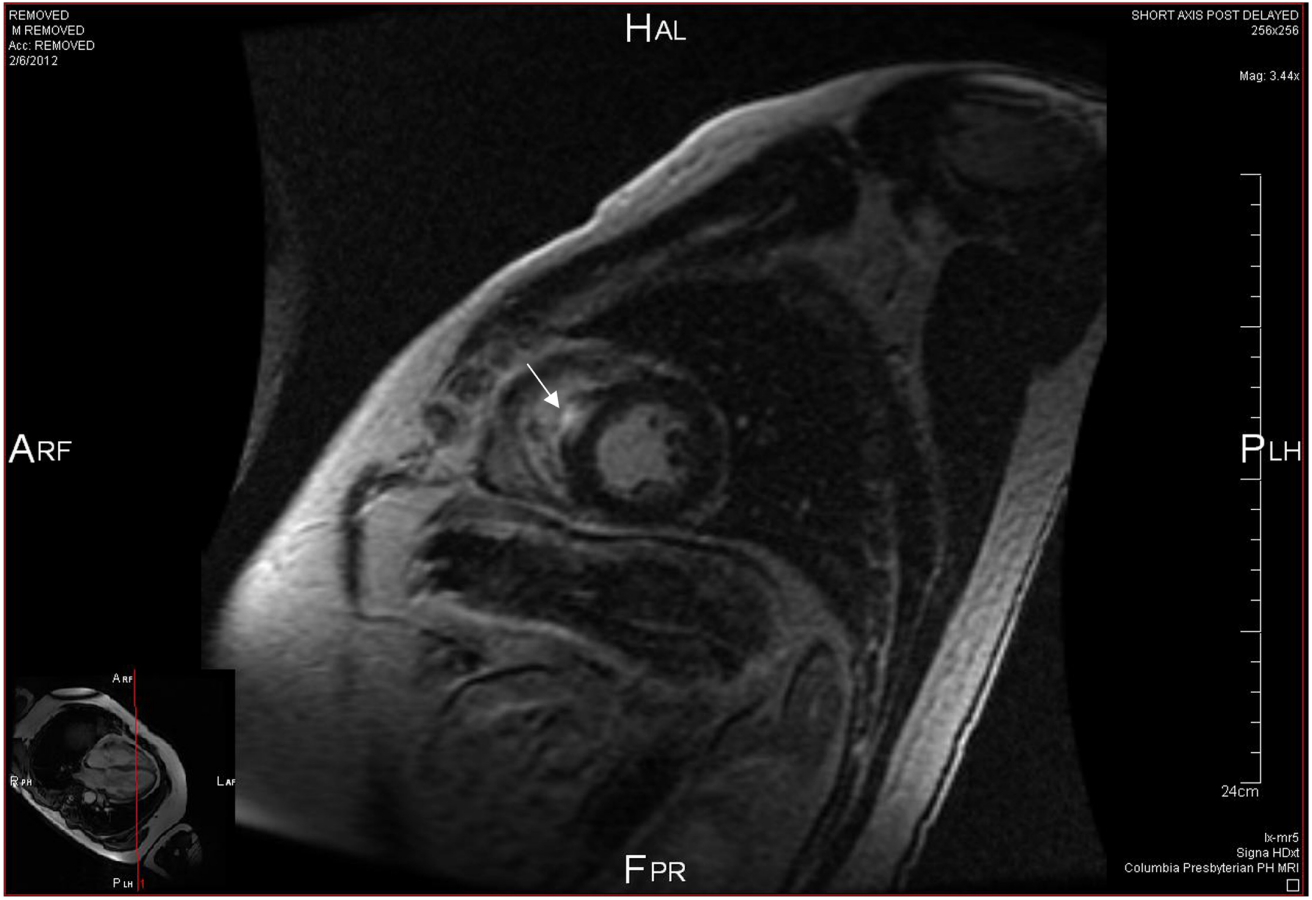
Cardiac MRI sagittal view at mid ventricle level showing focal late gadolinium enhancement (white arrow) in the interventricular septum.

Cardiac Magnetic Resonance imaging (CMR) is a multimodal, noninvasive assessment tool used for the evaluation of CS by demonstrating edema and scarring within the myocardium. While CMR can visualize structural abnormalities suggestive of CS such as ventricular septal wall thinning, ventricular aneurysm and local dyskinesia (17), the essential principle underlying CS detection by CMR is that of delayed postcontrast imaging (18). Delayed gadolinium contrast washout represents edema and inflammation in the acute setting and fibrous replacement in the chronic phase of CS relative to normal myocardial tissue (19, 20). CMR determines the presence of late gadolinium enhancement (LGE) in patterns characteristic of CS (Figure 1), most frequently in patchy, multifocal distributions seldom with subendocardial involvement. Subendocardial LGE is generally a sequela of ischemic heart disease infarct but can still be seen in CS (21). LGE findings most commonly involve the subepicardial right ventricular and basal left ventricular septal portions of the heart (17, 22). It is prudent to note that there are no diagnostic LGE patterns on CMR for CS, however. A recent meta-analysis has explicated the test characteristics of CMR. Of the studies included, 33 studies assessing the diagnostic accuracy of CMR and FDG-PET in CS have shown the sensitivity of CMR to be 95% with a specificity of 85% (20). LGE is an important prognostic marker and independent risk factor for death in CS. A prospective study following 155 patients with systemic sarcoidosis who underwent CMR for detection of possible cardiac disease reports a hazard ratio of 31.6 for death and aborted sudden cardiac death if LGE is present on imaging, which is greater than 30 times the ratios reported for LVEF or end-diastolic volume (23). The extent of LGE is also a sensitive marker of prognosis. Patients with significant LGE burden (greater than 20% of LV mass) were shown to have an increased risk of cardiac mortality, arrhythmia, hospitalization from heart failure, and absence of LV functional improvement following steroid therapy (24). The addition of T2 mapping to CMR allows for the identification of the acute inflammatory response in myocardial tissue and potentially allows for the early detection of subclinical CS (25, 26). Earlier identification of disease may predict impending clinical deterioration and help tailor responses to immunosuppressive therapies (26, 27). Coupled with LGE findings in more advanced disease, T2 mapping permits comprehensive CMR evaluation in the CS workup.

**Figure 2:**
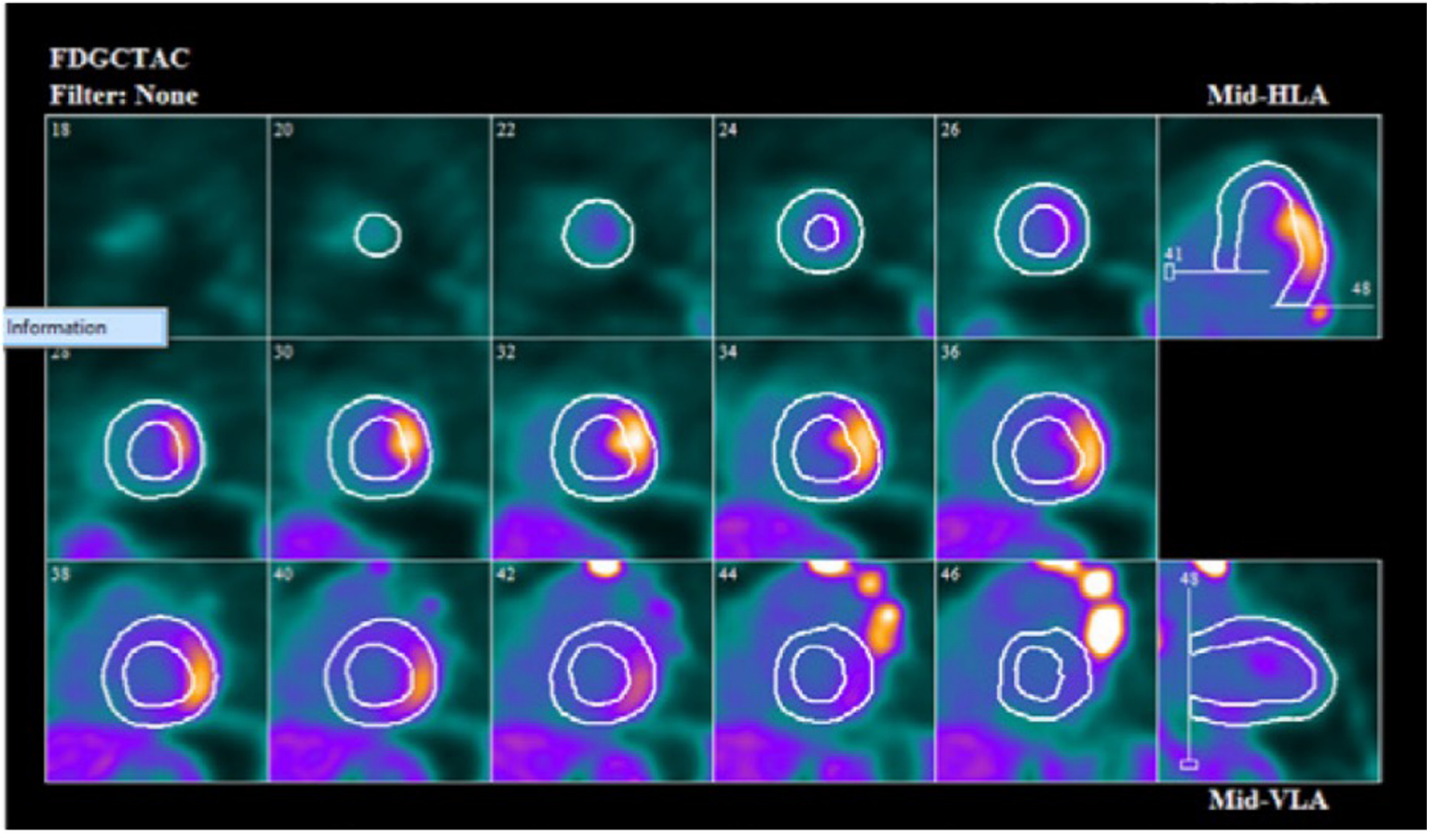
Cardiac F-18 FDG PET scan showing focal uptake of FDG in mid lateral wall on short axis, vertical long axis and horizontal long axis.

^18^F-flurodeoxyglycose positron emission tomography (FDG-PET) is an advanced imaging modality utilized in the diagnosis, prognostication and treatment monitoring of CS. The primary method of detecting CS by FDG-PET relies on identifying areas of increased ^18^F-FDG uptake in myocardial tissue which correspond to pathological cardiac inflammation (Figure 2). Inflamed sarcoid granulomatous tissue will readily take up glucose and its analogs (28), a process which can be mapped by multidimensional imaging and localize abnormal lesions. Pre-imaging preparation requires a high-fat, low-carbohydrate diet for 24 h prior to scanning to minimize dietary glucose–related competitive inhibition of ^18^F-FDG uptake (29). An area of abnormal ^18^F-FDG uptake corresponding to a known perfusion defect is a distinguishing feature seen in CS, known as a mismatch pattern (5). Myocardial scarring or focal reversible vasoconstriction in arterioles adjacent to sarcoid granulomas are posited to lead to perfusion defects (30). The sensitivity and specificity for FDG-PET were shown to be 84% and 83% respectively as per a recent meta-analysis evaluating the diagnostic performance of the test across 17 studies and close to 900 patients with suspected CS (31). A final consideration prior to performing FDG-PET would be to exclude significant coronary artery disease (CAD). Myocardial ischemia from underlying CAD can result in both abnormal perfusion and ^18^F-FDG uptake. Stress myocardial perfusion imaging would be of limited use given that resting perfusion defects may be attributable to either CS or CAD. The 2017 SNMMI/ASNC Expert Consensus Statement recommends CT coronary angiography or invasive angiography prior to FDG-PET to assess for anatomic stenoses once a patient's age and risk factors have been considered (32).

The preponderance of current available evidence confirms the prognostic value of FDG-PET. A contemporary meta-analysis performed by Bhatia et al. evaluated 40 studies with 495 participants to ascertain the prognostic significance of FDG-PET imaging in patients with suspected or diagnosed CS (33). Patients with abnormal ^18^F-FDG uptake had higher odds of major adverse cardiac events, including sustained ventricular tachycardia and sudden cardiac death (OR 3.12, CI 1.9–5.01 *p* < 0.00001) as compared to known or suspected CS patients with normal FDG-PET. Focal right ventricular uptake was shown to be an independent predictor of major adverse cardiac events (OR 5.24, CI 1.1–25.1, *p* = 0.04) (33, 34). The meta-analysis performed by Ahmed et al. showed odds ratios of 2.08 (CI 1.48–2.92) and 2.96 (CI 1.12–7.78) for abnormal LV and RV FDG-PET and major adverse cardiac events, respectively (35).

Quantification of ^18^F-FDG uptake by myocytes is possible with calculation of the standardized uptake value (SUV) (28), allowing for objective assessment of treatment response over time (36, 37). Flores et al. showed that SUV can also be used to predict future clinical outcomes. Poisson regression analysis revealed that SUV at the time of CS diagnosis has significant associations with total cardiac events. Although OR for total cardiac events with maximum SUV was 1.068 (95% CI 1.024–1.114, *P* = 0.002), patients with higher SUV, particularly in basal segments, are at an increased risk of cardiac events. These events include ventricular tachycardia, AICD and PPM placement, worsening ejection fraction and death (38). The study concurs with an earlier prospective study following 23 patients over 2 years who were treated with corticosteroids (91%), angiotensin-converting enzyme inhibitors/angiotensin-receptor blockers (78%), and beta-blockers (83%) (39). Longitudinal regression demonstrated a significant inverse linear relationship between maximum SUV and LVEF, with EF increasing 7.9% per SUV decrease by 10 g·ml(−1) (*P* = .008). This data emphasizes the developing prognostic importance of quantitative FDG-PET data in relation to LVEF. Of interest, the study was also able to identify treatment non-responders who experienced decreases in LVEF with standard therapies. This has real-world implications as it permits tailoring and escalation of therapy at an earlier stage if CS patients are not responding to the initial treatment regimen.

**Figure 3:**
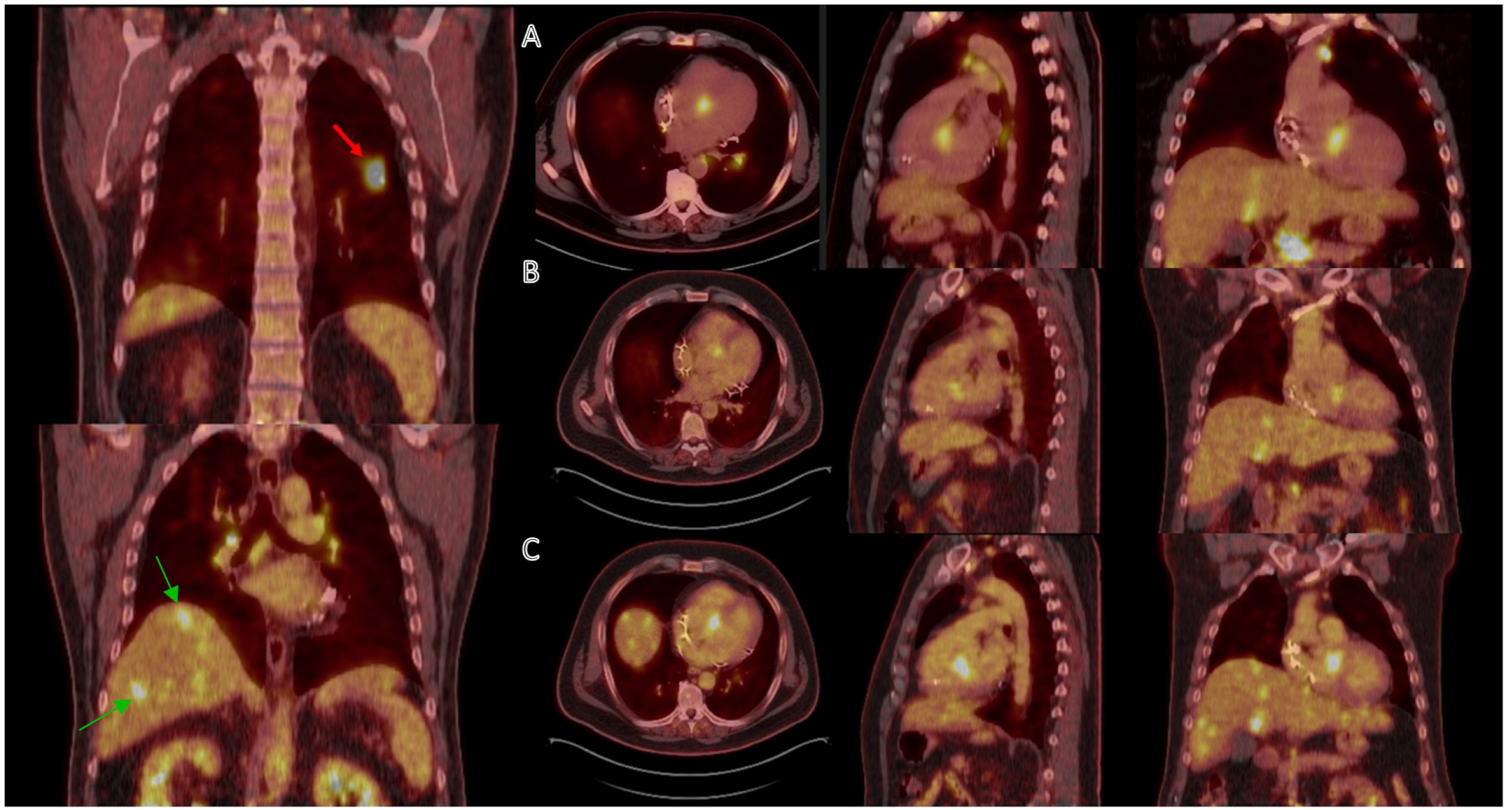
A 51-year-old man was found to have systemic sarcoidosis involving the lung (red arrow), liver (green arrows), and heart. **(A)** Before treatment, cardiac PET scan showed focal FDG uptake near the basal septum. **(B)** After 3 months of prednisone therapy, partial treatment response was achieved as evidenced by decreased FDG uptake in the myocardium. There was also a significant interval decrease in FDG-avid extra-cardiac lesions. **(C)** However, follow-up cardiac PET scan after a gradual 6-month prednisone taper demonstrated increasing FDG uptake in the myocardium and liver, suggesting relapse of sarcoidosis. Methotrexate was initiated.

Benefits of CMR relative to FDG-PET include no patient exposure to ionizing radiation or need for patients to adhere to a specialized ketogenic preparatory diet. CMR is more readily available than FDG-PET although absolute numbers of physicians providing CMR services remain limited (1.0% of radiologists and 0.2% of cardiologists) (40). CMR can assess cardiac structure, function and tissue characterization, rendering it ideal to assess for alternative infiltrative disorders or cardiomyopathies that may account for a patient's clinical presentation. CMR also has a substantially lower rate of nondiagnostic studies compared to FDG-PET, with the latter approaching a 15% diagnostic failure rate secondary to insufficient suppression of physiologic glucose uptake (41). FDG-PET, conversely, benefits from a lack of interference from motion artifact or the inability of a patient to breath-hold. FDG-PET is, moreover, the preferred imaging modality of choice in patients with implantable cardiac devices or those with severely reduced renal function (42). Although T2 mapping has made CMR more adept at identifying CS at earlier stages, FDG-PET remains more proficient in diagnosing active inflammation in early disease, thus affording the physician an opportunity to initiate prompt immunosuppression. FDG-PET is also preferred over CMR for monitoring treatment response over time as SUV allows for the quantification of disease activity (Figure 3). Finally, FDG-PET allows for detection of systemic sarcoidosis with concomitant full-body FDG imaging, a finding present in 97% of CS patients (43).

## 3 Management

Management of CS requires therapies targeting multiple aspects of the disease process. Active inflammation within the myocardium must be dampened with immunosuppressive agents and can be achieved by the use of corticosteroids, steroid-sparing agents, or a combination of both. Ventricular arrhythmia is treated with antiarrhythmics and ICD implantation can reduce the risk of sudden cardiac death in select patient populations. Left ventricular dysfunction can be managed with medical therapy to limit cardiac remodeling. These various avenues of treatment may be overlapping and occurring simultaneously. Every facet of CS management must be tailored to the individual, with particular focus on the clinical team's concern for disease activity, risk assessment and LV ejection fraction.

First-line treatment for CS involves nonspecific immunosuppression utilizing corticosteroids, which are initiated when evidence of active inflammation on EMB/PET/MRI and clear clinical signs and symptoms are manifested (6, 7, 44). A systematic review of more than 1,100 patients across 34 publications demonstrated that corticosteroids improve atrioventricular nodal conduction in 43% of patients and may promote left ventricular function recovery; the data on ventricular arrhythmias and mortality was too limited to draw any meaningful conclusions (45). There is some observational data to refute whether steroids have any benefit in patients with severe LV dysfunction (46, 47). The benefit of immunosuppression in subclinical disease in the absence of LV dysfunction has not yet been established. In these patients management decisions must be based on the presence of any extracardiac disease and the degree of active inflammation on advanced imaging. There are presently no standardized protocols or guidelines for the initiation and monitoring of patients with CS on corticosteroids. Lehtonen et al. in a recent clinical review has suggested tapering prednisone down by 5–10 mg every month until the patient is maintained on a dosage of 10 mg per day (5). Subsequently, corticosteroids would be discontinued at 12–16 months if there are no signs of disease activity. Serial annual follow-ups for 3–5 years with symptom evaluation, cardiac biomarkers, electrocardiogram and echocardiogram for left ventricular ejection fraction are recommended. The authors advise FDG-PET only if suspected relapse or treatment failure occurs, or if there are inconsistencies between clinical observations (5). This is in contrast to many institutes that perform routine FDG-PET to monitor disease activity and treatment response (48, 49). Pneumocystis Jirovecii prophylaxis with trimethoprim/sulfamethoxazole is encouraged once on corticosteroid doses greater than 20 mg daily (49). The Japanese Circulation Society recommends an initial prednisolone dose of 30 mg daily or 60 mg on alternate days for a 4-week period, followed by tapering of 5 mg monthly to reach a maintenance dose of 5–10 mg daily or 10–20 mg on alternate days by 6 months (7). Birnie et al. recommended starting with 30–40 mg of prednisone daily and tapering to 5–15 mg once an adequate treatment response was noted after 1 to 3 months. Treatment was continued for up to 12 months (50).

Corticosteroid sparing immunosuppressive therapies can be trialed to lessen the steroid burden or in the event of corticosteroid treatment failure, rapidly progressive heart failure, life-threatening arrhythmias or extensive inflammation on imaging. Rosenthal et al. demonstrated that low-dose prednisone (less than 10 mg) with either methotrexate or adalimumab is an effective maintenance therapy in patients after an initial response is confirmed (51). Such an approach would help to mitigate the unfavorable side-effects associated with chronic corticosteroid use. Methotrexate is the most widely used steroid-sparing agent in CS. Its common utilization as monotherapy or in combination with steroids in pulmonary sarcoidosis has been extrapolated to CS (52, 53). Vis et al. showed significant suppression of cardiac FDG uptake specifically in CS patients after 6 months of prednisone, methotrexate or combination therapy; there were no significant differences in clinical outcomes during follow-up over 24 months (54). This may be compared with other data suggesting improved outcomes with immunosuppressants utilized in combination with corticosteroids for the treatment of CS (55, 56). However, these selected studies are small and definitive conclusions cannot be drawn (44). Infliximab and adalimumab, both biologic anti-tumor necrosis (TNF) inhibitors, are third-line agents for CS and have shown promise in reducing cardiac inflammation when other treatment modalities have failed (48, 57). Anti-B-cell therapy with rituximab was shown to have a beneficial effect in a small case series involving 7 patients with refractory CS, conclusions from such a sample size are naturally equivocal (58). All biologic agents require comprehensive screening and vaccination for tuberculosis prior to initiation, in addition to close monitoring for infection and other complications.

Treatment for left ventricular dysfunction in CS should be initiated with guideline-directed medical therapies (GDMT) as well as immunosuppression. GDMT has been recognized to limit deleterious cardiac remodeling in heart failure and includes beta-blockers, angiotensin-converting enzyme inhibitors, angiotensin receptor blockers and neprilysin inhibitor–angiotensin receptor combinations. Mineralocorticoid receptor antagonists and sodium-glucose cotransporter-2 inhibitors are also within the GDMT framework. Left ventricular systolic dysfunction on presentation has been reported as an independent predictor of adverse outcomes and mortality in CS patients (49, 59). It is judicious to note that data regarding GDMT use specifically in CS is lacking and has been extrapolated from established data on patients with heart failure with reduced ejection fraction (60, 61).

Ventricular tachycardia (VT) is a recognized clinical manifestation of CS. Re-entry circuits developing in fibrotic and inflamed myocardial tissue may generate sustained ventricular tachycardia, which can be seen in up to 17% of patients with active disease as evidenced by a Finnish registry (62). The HRS consensus statement advises antiarrhythmic medication, most commonly either sotalol or amiodarone, after immunosuppression initiation for unresponsive VT (6). Catheter ablation is reserved for patients refractory to medical therapy. In a meta-analysis of 401 patients with refractory VT across 15 studies, the recurrence rate of VT after first ablation was 55% and 37% after multiple procedures (63). Of reassurance, there is observational data to show that catheter ablation is able to control VT storm associated with CS (64).

The risk of sudden cardiac death in patients with manifest clinical disease is 10% over five years (65). Expert societies are largely in concordance over indications for implantable-cardioverter defibrillators (ICD) in patients with CS. Both the HRS/ACC/AHA consortium and the ESC list the following as indications for ICD insertion in patients with CS: LVEF <35% despite immunosuppression, cardiac arrest, history of syncope compatible with arrhythmogenic etiology, history of sustained VT or inducible sustained VT at programmed electrical stimulation, and LVEF > 35% with extensive myocardial scarring on advanced imaging (66, 67). Given that many patients will meet ICD insertion indications at the time of presentation with CS (54), prudent clinical risk stratification and honest conversation with patients are of the utmost importance to ensure these individuals receive access to the appropriate secondary prevention tools.

## 4 Conclusion

In summary, diagnosing CS in the absence of endomyocardial biopsy necessitates the usage of advanced imaging techniques. Both CMR and FDG-PET are integral to the diagnostic workup of CS, however, neither modality can assure a diagnosis of cardiac sarcoidosis in isolation. The two imaging modalities should be viewed as complimentary given that they identify different pathological processes. FDG-PET is more adept at identifying the active inflammatory phase of CS and can help guide the initiation of treatment, whereas CMR best evaluates the chronic fibrotic phase of the disease via assessment of LGE. Indeed, hybrid CMR/FDG-PET was shown to be superior at detecting CS than both tests alone (68, 69). Management of CS consists of a stepwise approach utilizing corticosteroids, immunosuppressive medications and biologic agents. Antiarrhythmics should be used for VT unresponsive to immunosuppression. Specialized centers should be established to effectively manage CS patients; the diagnosis and care of these patients should be multidisciplinary in nature with experts in heart failure, cardiac imaging and electrophysiology involved. The results of the CHASM-CS randomized controlled trial are anticipated to expand current understanding concerning the effect of corticosteroid treatment on the clinical course of CS (70).

## References

[1] Drent M Crouser ED Grunewald J. Challenges of sarcoidosis and its management. N Engl J Med. (2021) 385(11):1018–32. 10.1056/NEJMra2101555 34496176

[2] Kandolin R Lehtonen J Airaksinen J Vihinen T Miettinen H Ylitalo K Cardiac sarcoidosis: epidemiology, characteristics, and outcome over 25 years in a nationwide study. Circulation. (2015) 131(7):624–32. 10.1161/CIRCULATIONAHA.114.011522 25527698

[3] Okada DR Bravo PE Vita T Agarwal V Osborne MT Taqueti VR Isolated cardiac sarcoidosis: a focused review of an under-recognized entity. J Nucl Cardiol. (2018) 25(4):1136–46. 10.1007/s12350-016-0658-1 27613395 PMC5540795

[4] Rosen NS Pavlovic N Duvall C Wand AL Griffin JM Okada DR Cardiac sarcoidosis outcome differences: a comparison of patients with *de novo* cardiac versus known extracardiac sarcoidosis at presentation. Respir Med. (2022) 198:106864. 10.1016/j.rmed.2022.106864 35550245

[5] Lehtonen J Uusitalo V Pöyhönen P Mäyränpää MI Kupari M. Cardiac sarcoidosis: phenotypes, diagnosis, treatment, and prognosis. Eur Heart J. (2023) 44(17):1495–510. 10.1093/eurheartj/ehad067 36924191 PMC10149532

[6] Birnie DH Sauer WH Bogun F Cooper JM Culver DA Duvernoy CS HRS expert consensus statement on the diagnosis and management of arrhythmias associated with cardiac sarcoidosis. Heart Rhythm. (2014) 11(7):1305–23. 10.1016/j.hrthm.2014.03.043 24819193

[7] Terasaki F Azuma A Anzai T Ishizaka N Ishida Y Isobe M JCS 2016 guideline on diagnosis and treatment of cardiac sarcoidosis- digest version. Circ J. (2019) 83(11):2329–88. 10.1253/circj.cj-19-0508 31597819

[8] Judson MA Costabel U Drent M Wells A Maier L Koth L The WASOG sarcoidosis organ assessment instrument: an update of a previous clinical tool. Sarcoidosis Vasc Diffuse Lung Dis. (2014) 31(1):19–27. 24751450

[9] Hussain K Shetty M. Cardiac sarcoidosis. In: StatPearls. Treasure Island, FL: StatPearls Publishing (2023). Available online at: http://www.ncbi.nlm.nih.gov/books/NBK578192/ (cited August 1, 2023) 35201720

[10] Trivieri MG Spagnolo P Birnie D Liu P Drake W Kovacic JC Challenges in cardiac and pulmonary sarcoidosis: a JACC state-of-the-art review. J Am Coll Cardiol. (2020) 76(16):1878–901. 10.1016/j.jacc.2020.08.042 33059834 PMC7808240

[11] Ezzeddine FM Kapa S Rosenbaum A Blauwet L Deshmukh AJ AbouEzzeddine OF Electrogram-guided endomyocardial biopsy yield in patients with suspected cardiac sarcoidosis and relation to outcomes. J Cardiovasc Electrophysiol. (2021) 32(9):2486–95. 10.1111/jce.15191 34314091

[12] Ji HL Xi NMS Mohan C Yan X Jain KG Zang QS Biomarkers and molecular endotypes of sarcoidosis: lessons from omics and non-omics studies. Front Immunol. (2024) 14:1342429. 10.3389/fimmu.2023.1342429/full 38250062 PMC10797773

[13] Kiko T Yoshihisa A Kanno Y Yokokawa T Abe S Miyata-Tatsumi M A multiple biomarker approach in patients with cardiac sarcoidosis. Int Heart J. (2018) 59(5):996–1001. 10.1536/ihj.17-695 30101857

[14] Kusano KF Satomi K. Diagnosis and treatment of cardiac sarcoidosis. Heart Br Card Soc. (2016) 102(3):184–90. 10.1136/heartjnl-2015-307877 26643814

[15] Di Stefano C Bruno G Arciniegas Calle MC Acharya GA Fussner LM Ungprasert P Diagnostic and predictive value of speckle tracking echocardiography in cardiac sarcoidosis. BMC Cardiovasc Disord. (2020) 20(1):21. 10.1186/s12872-019-01323-0 31959111 PMC6971954

[16] Burstow DJ Tajik AJ Bailey KR DeRemee RA Taliercio CP. Two-dimensional echocardiographic findings in systemic sarcoidosis. Am J Cardiol. (1989) 63(7):478–82. 10.1016/0002-9149(89)90323-8 2916434

[17] Smedema JP Snoep G van Kroonenburgh MPG van Geuns RJ Dassen WRM Gorgels APM Evaluation of the accuracy of gadolinium-enhanced cardiovascular magnetic resonance in the diagnosis of cardiac sarcoidosis. J Am Coll Cardiol. (2005) 45(10):1683–90. 10.1016/j.jacc.2005.01.047 15893188

[18] Patel MR Cawley PJ Heitner JF Klem I Parker MA Jaroudi WA Detection of myocardial damage in patients with sarcoidosis. Circulation. (2009) 120(20):1969–77. 10.1161/CIRCULATIONAHA.109.851352 19884472 PMC2778859

[19] Mahrholdt H Wagner A Judd RM Sechtem U Kim RJ. Delayed enhancement cardiovascular magnetic resonance assessment of non-ischaemic cardiomyopathies. Eur Heart J. (2005) 26(15):1461–74. 10.1093/eurheartj/ehi258 15831557

[20] Bravo PE. Cardiac MRI vs. PET for the Evaluation of Cardiac Sarcoidosis: Consider MRI First. Washington, DC: American College of Cardiology (2017). Available online at: https://www.acc.org/latest-in-cardiology/articles/2017/04/10/08/43/http%3a%2f%2fwww.acc.org%2flatest-in-cardiology%2farticles%2f2017%2f04%2f10%2f08%2f43%2fcardiac-mri-vs-pet (cited August 1, 2023)

[21] Watanabe E Kimura F Nakajima T Hiroe M Kasai Y Nagata M Late gadolinium enhancement in cardiac sarcoidosis: characteristic magnetic resonance findings and relationship with left ventricular function. J Thorac Imaging. (2013) 28(1):60–6. 10.1097/RTI.0b013e3182761830 23249970

[22] Vignaux O Dhote R Duboc D Blanche P Devaux JY Weber S Detection of myocardial involvement in patients with sarcoidosis applying T2-weighted, contrast-enhanced, and cine magnetic resonance imaging: initial results of a prospective study. J Comput Assist Tomogr. (2002) 26(5):762–7. 10.1097/00004728-200209000-00017 12439312

[23] Greulich S Deluigi CC Gloekler S Wahl A Zürn C Kramer U CMR imaging predicts death and other adverse events in suspected cardiac sarcoidosis. JACC Cardiovasc Imaging. (2013) 6(4):501–11. 10.1016/j.jcmg.2012.10.021 23498675

[24] Ise T Hasegawa T Morita Y Yamada N Funada A Takahama H Extensive late gadolinium enhancement on cardiovascular magnetic resonance predicts adverse outcomes and lack of improvement in LV function after steroid therapy in cardiac sarcoidosis. Heart Br Card Soc. (2014) 100(15):1165–72. 10.1136/heartjnl-2013-305187 24829369

[25] Crouser ED Ruden E Julian MW Raman SV. Resolution of abnormal cardiac MRI T2 signal following immune suppression for cardiac sarcoidosis. J Investig Med. (2016) 64(6):1148–50. 10.1136/jim-2016-000144 27354042

[26] O.Brien A. T2 mapping in myocardial disease: a comprehensive review. J Cardiovasc Magn Reson. (2022) 24(1):33. 10.1186/s12968-022-00866-0 35659266 PMC9167641

[27] Aquino G. Abstract 10664: quantitative T2 mapping to diagnose cardiac sarcoidosis and predict incident heart failure. Circulation. (2021) 144(Suppl_1). 10.1161/circ.144.suppl_1.10664

[28] Slart RHJA Glaudemans AWJM Lancellotti P Hyafil F Blankstein R Schwartz RG A joint procedural position statement on imaging in cardiac sarcoidosis: from the cardiovascular and inflammation & infection committees of the European association of nuclear medicine, the European association of cardiovascular imaging, and the American society of nuclear cardiology. J Nucl Cardiol. (2018) 25(1):298–319. 10.1007/s12350-017-1043-4 29043557

[29] Surasi DS Bhambhvani P Baldwin JA Almodovar SE O’Malley JP. ^18^F-FDG PET and PET/CT patient preparation: a review of the literature. J Nucl Med Technol. (2014) 42(1):5. 10.2967/jnmt.113.132621 24503347

[30] Kandolin R Ekström K Simard T Hibbert B Nery P Lehtonen J Spontaneous coronary artery dissection in cardiac sarcoidosis. Oxf Med Case Rep. (2019) 2019(5):omz033. 10.1093/omcr/omz033 PMC6544419 31198569

[31] Kim SJ Pak K Kim K. Diagnostic performance of F-18 FDG PET for detection of cardiac sarcoidosis; a systematic review and meta-analysis. J Nucl Cardiol. (2020) 27(6):2103–15. 10.1007/s12350-018-01582-y 30603894

[32] Chareonthaitawee P Beanlands RS Chen W Dorbala S Miller EJ Murthy VL Joint SNMMI–ASNC expert consensus document on the role of 18F-FDG PET/CT in cardiac sarcoid detection and therapy monitoring. J Nucl Med. (2017) 58(8):1341–53. 10.2967/jnumed.117.196287 28765228 PMC6944184

[33] Bhatia K Ramirez R Narasimhan B Walsh S Sud K Uberoi G Prognostic role of positron emission tomography in patients with known or suspected cardiac sarcoidosis. a systematic review and meta-analysis. Eur Heart J. (2020) 41(Supplement_2):ehaa946.0286. 10.1093/ehjci/ehaa946.0286

[34] Bekki M Tahara N Tahara A Sugiyama Y Maeda-Ogata S Honda A Localization of myocardial FDG uptake for prognostic risk stratification in corticosteroid-naïve cardiac sarcoidosis. J Nucl Cardiol. (2022) 29(5):2132–44. 10.1007/s12350-021-02684-w 34228338

[35] Ahmed A. Prognostic role of PET myocardial perfusion imaging in patients with cardiac sarcoidosis: a systematic review. Eur Heart J Cardiovasc Imaging. Oxford Academic.

[36] Bokhari S. FDG-PET is a Superior Tool in the Diagnosis and Management of Cardiac Sarcoidosis. Washington, DC: American College of Cardiology (2017). Available online at: https://www.acc.org/latest-in-cardiology/articles/2017/04/10/08/43/http%3a%2f%2fwww.acc.org%2flatest-in-cardiology%2farticles%2f2017%2f04%2f10%2f08%2f43%2ffdg-pet-is-a-superior-tool (cited August 1, 2023)

[37] Cabrera R Ananthasubramaniam K. Diagnosis, therapeutic response assessment, and detection of disease recurrence in cardiac sarcoidosis: integral role of cardiac PET. J Nucl Cardiol. (2016) 23(4):850–3. 10.1007/s12350-016-0399-1 26809438

[38] Flores RJ Flaherty KR Jin Z Bokhari S. The prognostic value of quantitating and localizing F-18 FDG uptake in cardiac sarcoidosis. J Nucl Cardiol. (2020) 27(6):2003–10. 10.1007/s12350-018-01504-y 30421379

[39] Osborne MT Hulten EA Singh A Waller AH Bittencourt MS Stewart GC Reduction in ^18^F-fluorodeoxyglucose uptake on serial cardiac positron emission tomography is associated with improved left ventricular ejection fraction in patients with cardiac sarcoidosis. J Nucl Cardiol. (2014) 21(1):166–74. 10.1007/s12350-013-9828-6 24307261

[40] Goldfarb JW Weber J. Trends in cardiovascular MRI and CT in the U.S. Medicare population from 2012 to 2017. Radiol Cardiothorac Imaging. (2021) 3(1):e200112. 10.1148/ryct.2021200112 33778651 PMC7977977

[41] Saric P Young KA Rodriguez-Porcel M Chareonthaitawee P. PET Imaging in cardiac sarcoidosis: a narrative review with focus on novel PET tracers. Pharmaceuticals. (2021) 14(12):1286. 10.3390/ph14121286 34959686 PMC8704408

[42] Writing group:, Document reading group:, EACVI Reviewers: This document was reviewed by members of the EACVI Scientific Documents Committee for 2014–2016 and 2016–2018. A joint procedural position statement on imaging in cardiac sarcoidosis: from the cardiovascular and inflammation & infection committees of the European association of nuclear medicine, the European association of cardiovascular imaging, and the American society of nuclear Cardiology. Eur Heart J Cardiovasc Imaging. (2017) 18(10):1073–89. 10.1093/ehjci/jex146 28984894

[43] Al-Hayja M. Cardiac sarcoidosis: the role of cardiac MRI and 18F-FDG-PET/CT in the diagnosis and treatment follow-up. Br J Cardiol. (2023) 30(1):7. 10.5837%2Fbjc.2023.007 37705835 10.5837/bjc.2023.007 PMC10495763

[44] Baughman RP Valeyre D Korsten P Mathioudakis AG Wuyts WA Wells A ERS clinical practice guidelines on treatment of sarcoidosis. Eur Respir J. (2021) 58(6):2004079. 10.1183/13993003.04079-2020 34140301

[45] Fazelpour S Sadek MM Nery PB Beanlands RS Tzemos N Toma M Corticosteroid and immunosuppressant therapy for cardiac sarcoidosis: a systematic review. J Am Heart Assoc. (2021) 10(17):e021183. 10.1161/JAHA.121.021183 34472360 PMC8649244

[46] Chiu CZ Nakatani S Zhang G Tachibana T Ohmori F Yamagishi M Prevention of left ventricular remodeling by long-term corticosteroid therapy in patients with cardiac sarcoidosis. Am J Cardiol. (2005) 95(1):143–6. 10.1016/j.amjcard.2004.08.083 15619415

[47] Wand AL Pavlovic N Duvall C Rosen NS Chasler J Griffin JM Effect of corticosteroids on left ventricular function in patients with cardiac sarcoidosis. Am J Cardiol. (2022) 177:108–15. 10.1016/j.amjcard.2022.04.051 35701237

[48] Gilotra NA Wand AL Pillarisetty A Devraj M Pavlovic N Ahmed S Clinical and imaging response to tumor necrosis factor alpha inhibitors in treatment of cardiac sarcoidosis: a multicenter experience. J Card Fail. (2021) 27(1):83–91. 10.1016/j.cardfail.2020.08.013 32889044 PMC8350936

[49] Giblin GT Murphy L Stewart GC Desai AS Di Carli MF Blankstein R Cardiac sarcoidosis: when and how to treat inflammation. Card Fail Rev. (2021) 7:e17. 10.15420/cfr.2021.16 34950507 PMC8674699

[50] Birnie DH Nery PB Ha AC Beanlands RSB. Cardiac sarcoidosis. J Am Coll Cardiol. (2016) 68(4):411–21. 10.1016/j.jacc.2016.03.605 27443438

[51] Rosenthal DG Parwani P Murray TO Petek BJ Benn BS De Marco T Long-term corticosteroid-sparing immunosuppression for cardiac sarcoidosis. J Am Heart Assoc Cardiovasc Cerebrovasc Dis. (2019) 8(18):e010952. 10.1161/jaha.118.010952 PMC6818011 31538835

[52] Goljan-Geremek A Bednarek M Franczuk M Puścińska E Nowiński A Czystowska M Methotrexate as a single agent for treating pulmonary sarcoidosis: a single centre real-life prospective study. Pneumonol Alergol Pol. (2014) 82(6):518–33. 10.5603/piap.2014.0069 25339562

[53] Isshiki T Yamaguchi T Yamada Y Maemura K Makita K Takeshima H Usefulness of low-dose methotrexate monotherapy for treating sarcoidosis. Intern Med Tokyo Jpn. (2013) 52(24):2727–32. 10.2169/internalmedicine.52.0976 24334575

[54] Vis R Mathijssen H Keijsers RGM van de Garde EMW Veltkamp M Akdim F Prednisone vs methotrexate in treatment naïve cardiac sarcoidosis. J Nucl Cardiol. (2023) 30(4):1543–53. 10.1007/s12350-022-03171-6 36640249

[55] Nagai S Yokomatsu T Tanizawa K Ikezoe K Handa T Ito Y Treatment with methotrexate and low-dose corticosteroids in sarcoidosis patients with cardiac lesions. Intern Med Tokyo Jpn. (2014) 53(5):427–33. 10.2169/internalmedicine.53.0794 24583430

[56] Ballul T Borie R Crestani B Daugas E Descamps V Dieude P Treatment of cardiac sarcoidosis: a comparative study of steroids and steroids plus immunosuppressive drugs. Int J Cardiol. (2019) 276:208–11. 10.1016/j.ijcard.2018.11.131 30527995

[57] Bakker ALM Mathijssen H Azzahhafi J Swaans MJ Veltkamp M Keijsers RGM Effectiveness and safety of infliximab in cardiac sarcoidosis. Int J Cardiol. (2021) 330:179–85. 10.1016/j.ijcard.2021.02.022 33582196

[58] Elwazir M Krause ML Bois JP Christopoulos G Kendi AT Cooper JLT Rituximab for the treatment of refractory cardiac sarcoidosis: a single-center experience. J Card Fail. (2022) 28(2):247–58. 10.1016/j.cardfail.2021.07.008 34320381

[59] Yazaki Y Isobe M Hiroe M Morimoto S Hiramitsu S Nakano T Prognostic determinants of long-term survival in Japanese patients with cardiac sarcoidosis treated with prednisone. Am J Cardiol. (2001) 88(9):1006–10. 10.1016/S0002-9149(01)01978-6 11703997

[60] Gilotra N Okada D Sharma A Chrispin J. Management of cardiac sarcoidosis in 2020. Arrhythmia Electrophysiol Rev. (2020) 9(4):182–8. 10.15420/aer.2020.09 PMC7788397 33437485

[61] Heidenreich PA Bozkurt B Aguilar D Allen LA Byun JJ Colvin MM 2022 AHA/ACC/HFSA guideline for the management of heart failure: a report of the American College of Cardiology/American Heart Association joint committee on clinical practice guidelines. Circulation. (2022) 145(18):e895–1032. 10.1161/cir.0000000000001063 35363499

[62] Ekström K Lehtonen J Nordenswan HK Mäyränpää MI Räisänen-Sokolowski A Kandolin R Sudden death in cardiac sarcoidosis: an analysis of nationwide clinical and cause-of-death registries. Eur Heart J. (2019) 40(37):3121–8. 10.1093/eurheartj/ehz428 31230070

[63] Adhaduk M Paudel B Liu K Ashwath M Giudici M. Meta-analysis of catheter ablation outcomes in patients with cardiac sarcoidosis refractory ventricular tachycardia. Am J Cardiol. (2022) 174:136–42. 10.1016/j.amjcard.2022.03.038 35504741

[64] Siontis KC Santangeli P Muser D Marchlinski FE Zeppenfeld K Hoogendoorn JC Outcomes associated with catheter ablation of ventricular tachycardia in patients with cardiac sarcoidosis. JAMA Cardiol. (2022) 2:175–83. 10.1001/jamacardio.2021.4738 PMC8600457 34787643

[65] Nordenswan HK Pöyhönen P Lehtonen J Ekström K Uusitalo V Niemelä M Incidence of sudden cardiac death and life-threatening arrhythmias in clinically manifest cardiac sarcoidosis with and without current indications for an implantable cardioverter defibrillator. Circulation. (2022) 146(13):964–75. 10.1161/CIRCULATIONAHA.121.058120 36000392 PMC9508990

[66] Al-Khatib SM Stevenson WG Ackerman MJ Bryant WJ Callans DJ Curtis AB 2017 AHA/ACC/HRS guideline for management of patients with ventricular arrhythmias and the prevention of sudden cardiac death: a report of the American College of Cardiology/American Heart Association task force on clinical practice guidelines and the heart rhythm society. J Am Coll Cardiol. (2018) 72(14):e91–220. 10.1016/j.jacc.2017.10.054 29097296

[67] Zeppenfeld K Tfelt-Hansen J de Riva M Winkel BG Behr ER Blom NA 2022 ESC guidelines for the management of patients with ventricular arrhythmias and the prevention of sudden cardiac death. Eur Heart J. (2022) 43(40):3997–4126. 10.1093/eurheartj/ehac262 36017572

[68] Wicks EC Menezes LJ Barnes A Mohiddin SA Sekhri N Porter JC Diagnostic accuracy and prognostic value of simultaneous hybrid 18F-fluorodeoxyglucose positron emission tomography/magnetic resonance imaging in cardiac sarcoidosis. Eur Heart J Cardiovasc Imaging. (2018) 19(7):757–67. 10.1093/ehjci/jex340 29319785

[69] Greulich S. Hybrid cardiac magnetic resonance/fluorodeoxyglucose positron emission tomography to differentiate active from chronic cardiac sarcoidosis. JACC Cardiovasc Imaging. (2022) 15(3):445–56. 10.1016/j.jcmg.2021.08.018 34656480

[70] Ottawa Heart Institute Research Corporation. Cardiac Sarcoidosis Multi-Center Randomized Controlled Trial. Ontario, Canada: Ottawa Heart Institute Research Corporation (2023). Report No.: NCT03593759. Available online at: https://clinicaltrials.gov/study/NCT03593759, clinicaltrials.gov (cited August 22, 2023)

